# Analysis of factors associated with substandard nutrition in critically ill patients and the effect of applying enteral nutrition nursing model based on evidence-based medicine: A retrospective observational study

**DOI:** 10.1097/MD.0000000000044231

**Published:** 2025-12-12

**Authors:** Jie Wang, Jianhui Wang, XiaoXia Ye, Qingqin Lu, Ya Liu

**Affiliations:** a Department of Central Guardianship Unit, The Fifth People’s Hospital of Shanghai, Shanghai, China.

**Keywords:** critical illness, enteral nutrition, evidence-based, influencing factors, nutrition

## Abstract

The aim was to study the factors related to substandard nutrition in critically ill patients and the application effect of enteral nutrition nursing model based on evidence-based medicine. A total of 137 cases of critically ill patients treated in our hospital from January 2023 to January 2024 were clinically selected as the research subjects, and they were divided into the standardized group and the non-standardized group based on the patients’ enteral nutrition. We collected and compared the clinical data of patients in the 2 groups, analyzed the covariance of the difference indicators, included the indicators without covariance problems in the logistic regression model to analyze the factors related to the patients’ enteral nutrition not meeting the standard, constructed the clinical prediction model, and presented it as a visualization with a column line diagram, and assessed the predictive ability of the column line diagram model through internal validation of the drawing of the subject characteristics curve (receiver operating characteristic curve). The evidence-based medicine enteral nutrition care model was implemented for the patients, and its clinical application effect was analyzed. There were significant differences (*P* < .05) in the comparison of Glasgow Coma score, modified Nutritional Risk in Critical Illness score, catecholamines, feeding intolerance, and Acute Physiology and Chronic Health Evaluation II scores between the 2 groups. All difference variables were analyzed for variance inflation factor covariance using the R language (R package: logreg6.2.0), and the variance inflation factor of each difference variable was ≤10 with tolerance ≥0.1, so there was no problem of covariance, and the above indexes could be included in the logistic regression model, and the results found that all of the above indexes were the independent influences on nutritional nonattainment in critically ill patients (*P* < .05). A column-line graph prediction model was established, and the area under the curve value in the receiver operating characteristic curve was 0.950 with a 95% CI of (0.910–0.989), thus indicating that this clinical prediction model has a good degree of risk prediction. The patients’ various nutritional indexes and enteral nutrition tolerance rate after implementation were better than before implementation (*P* < .05). Glasgow Coma score, modified Nutritional Risk in Critical Illness score, catecholamines, feeding intolerance, and Acute Physiology and Chronic Health Evaluation II Score are all independent influencing factors affecting the occurrence of enteral nutrition substandard in critically ill patients. The above indicators can be used to screen high-risk groups for the clinic, based on the evidence-based medicine enteral nutrition nursing care model and can effectively improve the nutritional status of the patient and the degree of nutritional tolerance. It provides a theoretical basis for clinical practice.

## 1. Introduction

Critically ill patients usually refers to a category of patients whose condition is acute in onset, critical and rapid in change, and a slight inadvertence may result in irreparable consequences.^[1]^ According to relevant data, due to disease attack or trauma and other impacts, critically ill patients often have a series of physiopathological and metabolic changes, often accompanied by digestive system dysfunction, resulting in difficulty in maintaining a normal diet, reduced energy intake, and then fall into the plight of nutritional deficiencies.^[2,3]^ This situation not only weakens the immune defense of patients, leading to decreased immune function and increased risk of infection, but also prolongs hospitalization time, increases medical costs, and even affects the survival rate of patients, posing a serious challenge to the process of patient recovery.^[4]^ Therefore, it is particularly urgent and far-reaching to actively analyze the relevant factors affecting the substandard enteral nutrition of critically ill patients, and explore and apply efficient enteral nutrition nursing models relying on the solid cornerstone of evidence-based medicine.

Xie et al^[5]^ indicated that enteral nutrition, as the preferred nutritional support method, is highly valued by clinics because it can better maintain intestinal function, promote intestinal function recovery, reduce systemic inflammatory response, lower infection risk, shorten hospitalization time, and improve overall recovery effect, but the implementation of enteral nutrition in actual application faces many challenges, especially the problem of the target rate of enteral nutrition, which becomes a key factor affecting the prognosis of patients. It has become one of the key factors affecting patients’ prognosis. On the one hand, the pathophysiological changes of the disease itself, such as high metabolic state, accelerated protein catabolism and fat metabolism disorders, significantly increase the body’s demand for energy and nutrients; on the other hand, the intestinal tract, as the main place of nutrient absorption, impaired function directly impedes the effective uptake of nutrients, coupled with the many limitations in the process of treatment, such as fasting, surgical interventions, further exacerbating the nutritional deficiency of patients. On the other hand, the impaired function of the intestine as the main site of nutrient absorption directly hinders the effective intake of nutrients, coupled with many restrictions during the treatment process, such as fasting and surgical interventions, etc, which further exacerbate the nutritional deficiencies of the patients.^[6,7]^ Robinson et al^[8]^ scholars stated that an evidence-based medicine-based enteral nutrition care model has emerged to address the problem of substandard enteral nutrition. Evidence-based medicine emphasizes the application of well-designed and executed research evidence to optimize decision-making to improve the quality of care and patient satisfaction, following the principle of applying the best available research rationale carefully, accurately, and judiciously, and obtaining best-practice guidelines and recommendations through systematic literature searches and synthesis of evidence to ensure that the care provided is based on the latest and most reliable scientific evidence.^[9,10]^ This suggests that actively analyzing the factors associated with nutritional substandards in critically ill patients and implementing a model of enteral nutrition care based on evidence-based medicine may have better efficacy, but few clinically relevant studies have been reported.

Based on this, we are committed to constructing a comprehensive and in-depth analysis framework, firstly, through a systematic review of existing clinical practice cases, carefully combing the multiple factors of nutritional substandard in critically ill patients, including but not limited to the characteristics of the disease, changes in physiological function, etc, and then explore the potential impact of the evidence-based enteral nutrition nursing model on critically ill patients, in order to provide a set of operable and replicable nursing models for the nutritional management of critically ill patients. In order to provide a set of operable and replicable nursing models for nutritional management of critically ill patients, we will promote the in-depth integration of enteral nutrition theory and practice, promote the continuous optimization and innovation of nutritional support strategies in the field of critical care medicine, and provide theoretical support for clinical practice.

## 2. Methodology

### 2.1. Statement of ethics

This study was approved by the Institutional Review Board of The Fifth People’s Hospital of Shanghai. Requirement for informed consent was waived because only de-identified retrospective data were used. Given that this study was retrospective and only de-identified patient data were used, informed consent was not required as there were no risks or adverse effects to patients. This waiver is in line with regulatory and ethical guidelines related to retrospective studies.

### 2.2. Study design

One hundred thirty-seven critically ill patients treated at our hospital between January 2023 and January 2024 were retrospectively selected. Patient data were obtained from the medical record system, and patients were categorized into standardized and non-standardized groups based on their different enteral nutritional status.

### 2.3. Inclusion criteria

Inclusion criteria: age > 18 years; compliance with the Acute Physiology and Chronic Health Evaluation II (APACHE II)^[11]^ score ≥ 8; complete clinical data; those who do not have concomitant neurological diseases or cognitive dysfunction; those who are not breastfeeding or pregnant.

### 2.4. Exclusion criteria

Long-term use of hormones or immunosuppressants; those who were undergoing radiotherapy, chemotherapy, or special treatment for malignant tumors; irreversible terminal status; those who died, were discharged from the hospital, or were transferred to the general wards; and those who participated in other clinical studies.

### 2.5. Data collection

Patient data were collected through the medical record system, including age, gender (male/female), body mass index, hypertension in accordance with “The Japanese Society of Hypertension Guidelines for Self-monitoring of Blood Pressure at Home (Second Edition)^[12]^ (yes/no), hyperlipidemia in accordance with the Report of the Japan Atherosclerosis Society (JAS) Guideline for Diagnosis and Treatment of Hyperlipidemia in Japanese Adults,^[13]^ and the number of patients with hyperlipidemia in accordance with the Japanese Society of Hypertension Guidelines for Self-monitoring of Blood Pressure at Home (Second Edition).^[14]^ Japanese adults”^[13]^ (yes/no), diabetes mellitus in accordance with “Application of the Chinese Expert Consensus on Diabetes Classification in clinical practice”^[14]^ (yes/no), and diabetes mellitus (in accordance with the “Guideline for Diagnosis and Treatment of Hyperlipidemia in Japanese Adults”^[15]^ (yes/no). Diabetes Classification in clinical practice^[14]^ (yes/no), smoking history (number of cigarettes smoked per day > 1, and the duration of continuous time > 1 year or the time of quitting smoking < 1 year) (yes/no), history of alcohol consumption (yes/no) (the amount of alcohol consumed per day > 1 drinking volume, and the duration of time > 1 year or the time of quitting drinking < 1 year [1 drinking volume is 45 mL white wine/360 mL beer/120 mL wine]), analgesic and sedative drugs (yes/no), catecholamines (yes/no), and other medications (yes/no), as well as other medications (yes/no), catecholamines (yes/no), feeding intolerance (meeting the criteria related to the definition of feeding intolerance in the 2016 American Society for Parenteral and Enteral Nutrition^[15]^ Guidelines for Nutritional Support of Critically Ill Patients) (yes/no), mechanical ventilation (yes/no), gastric residual volume (<100 mL/≥100 mL), and continuous renal replacement therapy (yes/no).

### 2.6. State of consciousness

The patients’ consciousness was assessed according to Glasgow Coma score (GCS),^[16]^ including eye-opening response, verbal response and motor response in 3 dimensions, with a total score of 15, 15 indicating that the patients were able to spontaneously open their eyes, communicate, and move, and 3 indicating that the patients were unresponsive to stimuli, and the scores were positively correlated with the degree of conscious awareness.

### 2.7. Nutritional risk status

Evaluation was based on the modified Nutritional Risk in Critical Illness Score (mNU-TRIC),^[17]^ including age (0–2 points), number of comorbidities (0–1 points), time of admission and transfer to the intensive care unit (ICU) (0–1 points), assessment of sepsis-related organ failure (0–2 points), APACHE II (0–3 points), with the total score being the sum of the items (0–9 points), and the higher the score indicated that the higher the score, the higher the risk of malnutrition.

### 2.8. Acute physiological and chronic health scores

Assessment was performed according to APACHE II, consisting of acute physiology (including 12 physiological parameters, such as body temperature, heart rate, respiratory rate, blood pressure, oxygen saturation, blood gas analysis results, etc, and each parameter was assigned different scores according to its degree of deviation from the normal value), age (different scores were assigned according to the patient’s age, and the older the patient was, the higher the score was), and chronic health status (according to the patient’s chronic health status) (different scores are assigned according to the patient’s chronic health status, including whether there is severe chronic organ failure or immunosuppression). The final result is the sum of the 3 parts ranging from 0 to 71 points, with the score being proportional to the severity of the disease.

### 2.9. Nutritional compliance

According to the European Society for Parenteral and Enteral Nutrition^[18]^ guidelines for enteral nutrition, the daily amount of enteral nutrition required by patients is 25 kcal/(kg-d) (1 kcal = 4–18 kJ). When the patient is under stress, 60% of the patient’s target feeding rate can be given. 60% of the target feeding on day 3 of enteral nutrition was used as the cutoff, with ≥60% of the target feeding as the compliance group and <60% as the substandard feeding group.

### 2.10. Implementation of enteral nutrition nursing model based on evidence-based medicine

*Evidence-based issues*: A nurse manager and 5 specialized nurses were selected to search relevant databases with the Chinese keywords “enteral nutrition intolerance, enteral nutrition tolerance, early enteral nutrition intervention, enteral nutrition for critically ill patients, intensive care nursing,” etc, and to formulate an implementation plan by taking into account the development of the disease, the degree of physical tolerance and the actual needs. implementation program. *Evidence-based support*: according to the type of literature to take the corresponding evaluation standards, such as clinical practice, guidelines and other related evaluation standards can be taken APACHE II score, systematic evaluation, expert consensus and expert opinion can be taken to the corresponding evaluation standards of the Evidence-Based Health Care Center for evaluation, to carry out the actual analysis of the condition and countermeasures to intervene. *Evidence-based nursing measures*: Condition assessment, assessment of enteral nutrition feeding tolerance in critically ill patients, including stool examination, radiological assessment, glycemic control status and complaint symptoms, to avoid inappropriate cessation of enteral nutrition, and to assess feeding intolerance of patients through the Nutrition Intolerance Assessment Scale. *Nutritional management*: enteral nutrition preparation is enriched with glutamyl amino acid and chain fat carnitine to improve the tolerance of enteral nutrition feeding in critically ill patients; semisolid agent can be given before enteral nutrition infusion to improve the tolerance of enteral nutrition support; short peptide enteral nutrition preparation is recommended for enteral nutrition intolerance in critically ill patients; intravenous nutrition support is provided for those who are completely disabled. *Feeding program*: gastrointestinal nutrition intolerance, gastric discharge obstruction or risk of aspiration patients, can take the post-pyloric feeding route, such as nasoenteric tube, etc, the nasal feeding rate will be controlled to 15 to 50 mL/h, and then increased by 10 to 50 mL/h for 6 days, gradually increased to the target feeding rate. For critically ill patients who are intolerant to early enteral nutritional support, a nourishing feeding regimen (41.8–82.9 kJ/h) can be adopted for 6 days. For enteral nutritional support, the temperature of the nutrient solution is maintained at 38 to 42 °C, and it is manually pushed intermittently or infused by intermittent nutritional pumps, and the nasogastric tube is left in place to feed transgastrically. *Gastric residual volume monitoring*: monitor the gastric residual volume every 4 hours, or perform bedside ultrasound to monitor the gastric residual volume to avoid intolerance or aspiration of enteral nutrition. *Drug application*: use intravenous erythromycin at a dose of 100 to 250 mg 3 times a day for 2 to 4 days. Use erythromycin and metoclopramide in combination to promote emptying and improve the tolerance of enteral nutrition support in critically ill patients, and use cholecystokinin antagonist as an alternative drug for enteral nutrition intolerance. *Intra-abdominal pressure monitoring and management*: 2 consecutive monitoring to ensure that the gastric residual volume > 250 mL, intra-abdominal pressure abnormally increased in patients with severe pancreatitis, recommended the use of indirect measurement to monitor the patient’s intravesical pressure, adjusting the enteral nutrition support program. *Enteral nutrition Chinese medicine therapy*: to take Chinese medicine, acupuncture, laxative enema, etc, to improve the early enteral nutrition tolerance in patients with severe diseases; the presence of abdominal distension, nausea, vomiting in severe patients can be taken to take Chengqi Tang, Houpao Exhaustion Combination, ShengDaHuang, etc, for internal administration to regulate the qi and internal organs, but also to take acupoints such as acupuncture or abdominal massage, etc, and if the patients are intolerant of enteral nutrition, it is recommended that the use of the annexed zi li zhong pills combined with shenque point moxibustion.

### 2.11. Nutritional status

Collect patients’ morning fasting venous blood 5 mL, use centrifuge (model: LL900, manufacturer: Luoyang Hongshi Machinery Equipment Co., Ltd., Luoyang, Henan Province, China), 3000 r/min, centrifugation for 10 minutes, separate the patient’s serum, and place the sample in −80 °C environment for storage. The patient’s albumin (ALB), total protein (TP), prealbumin (PA) and hemoglobin (Hb) indexes were detected by using automatic blood biochemistry analyzer model: BOKE BK-200, manufacturer: Shandong BOKE Bio-industry Co., Jinan, Shandong Province, China.

### 2.12. Observation indicators

Collect the occurrence of patients’ enteral nutrition substandard, compare the clinical data of the standard group and the nonstandard group, conduct covariance analysis for the indicators of differences in single-factor analysis, conduct multifactorial analysis for the indicators without covariance problems, analyze the factors affecting the occurrence of patients’ nutritional substandard, and then construct the prediction model of the column line graph and validate it. And compare the nutritional indicators and enteral nutrition intolerance of patients before and after the implementation of the evidence-based medicine enteral nutrition care model.

### 2.13. Statistical processing

Statistical analysis was performed using IBM SPSS 27.0 (IBM Corp., Armonk), and the measurement data that conformed to normal distribution were expressed as (±s), *t* test, and those that did not conform to normal distribution used M (Q1,Q2), *Z* test; the count data, expressed as n, *x*² test, and the difference was considered statistically significant at *P* < .05. The indicators of differences were assigned as independent variables and the covariance between the indicators was calculated, and the indicators that did not have covariance problems with variance inflation factor ≤ 10 and tolerance ≥ 0.1 were included in the calculation of the logistic regression model to analyze the factors related to the occurrence of nutritional substandard of the patients, and the statistically significant variables in the regression analysis were used as the predictors, and the R language of the x64.4.1.3 version was taken and the Exogenous packages were adopted to draw column line graphs, and subject work characteristic curves (receiver operating characteristic [ROC] curves) were constructed to internally validate the column line graph risk prediction model. The manuscript adheres to the STROBE guideline; the completed checklist is provided as Checklist S1, Supplemental Digital Content, https://links.lww.com/MD/P904.

## 3. Results

### 3.1. Unifactorial analysis of the occurrence of nutritional substandard in critically ill patients

According to the records of medical record system, the occurrence of nutritional substandard in 137 critically ill patients was 47 patients, accounting for 34.31%, which was classified into the non-attainment group, and 90 patients with nutritional standard, accounting for 65.69%, which was classified into the attainment group. Patients in the non-attainment and attainment groups had GCS score (9.00 [6.00, 12.00] vs 13.00 [11.00, 14.00]) points, mNU-TRIC score (3.00 [2.00, 4.00] vs 2.00 [2.00, 2.00]) points, catecholamines ([yes/no], 15/32 vs 10/80), feeding intolerance ([yes/no], 11/36 vs 5/85), and APACHE II score (22.88 ± 5.31 vs 16.66 ± 5.24) points were compared with a statistically significant difference (*x*²/*t*/*Z* = 4.622, 5.191, 8.958, 9.536, and 6.566, *P* < .05), as shown in Table 1.

**Table 1:** Single factor analysis of the occurrence of malnutrition in severe patients.

Index		Failure group (n = 47)	Target group (n = 90)	*x²/t/Z*	*P*
Age (years)		68.55 ± 7.49	68.24 ± 7.56	0.229	.820
Sex (n)	Male	28	50	0.203	.652
	Female	19	40		
Body mass index (kg/m²)		22.49 ± 2.31	22.37 ± 2.29	0.290	.772
Hypertension (n)	Have	10	10	2.559	.110
	No	37	80		
Hyperlipemia (n)	Have	5	7	0.316	.574
	No	42	83		
Diabetes (n)	Have	7	9	0.717	.397
	No	40	81		
Smoking history (n)	Have	22	31	1.990	.158
	No	25	59		
Drinking history (n)	Have	17	30	0.110	.740
	No	30	60		
GCS score (score)		9.00 (6.00, 12.00)	13.00 (11.00, 14.00)	4.622	<.001
mNU-TRIC Score (score)		3.00 (2.00, 4.00)	2.00 (2.00, 2.00)	5.191	<.001
Analgesic sedative (n)	Have	28	45	1.137	.286
	No	19	45		
Catecholamines (n)	Have	15	10	8.958	.003
	No	32	80		
Feeding intolerance (n)	Have	11	5	9.536	.002
	No	36	85		
APACHE II score		22.88 ± 5.31	16.66 ± 5.24	6.566	<.001
Mechanical ventilation (n)	Have	18	25	1.587	.208
	No	29	65		
Gastric residual volume (n)	<100 mL	31	69	1.796	.180
	≥100 mL	16	21		
Continuous renal replacement therapy (n)	Have	13	17	1.389	.39
	No	34	73		

APACHE II = Acute Physiology and Chronic Health Evaluation II, GCS = Glasgow Coma score, mNU-TRIC = modified Nutritional Risk in Critical Illness Score.

### 3.2. Analysis of covariance

The camp of critically ill patients was taken as the dependent variable (0 = attained, 1 = not attained), and the GCS score (measured value), mNU-TRIC score (measured value), catecholamines (0 = none, 1 = yes), feeding intolerance (0 = none, 1 = yes), and APACHE II score (measured value) were taken as the independent variables. The covariance results revealed that none of the above indicators had covariance problems (variance inflation factor ≤ 10, tolerance ≥ 0.1), and the above indicators could be included in the logistic regression model, as shown in Table 2.

**Table 2:** Collinearity analysis.

Influencing factor	VIF value	Tolerance
GCS score	1.087	0.920
mNU-TRIC Score	1.086	0.921
Catecholamines	2.519	0.397
Feeding intolerance	2.522	0.396
APACH-II Score	1.136	0.880

GCS = Glasgow Coma score, mNU-TRIC = modified Nutritional Risk in Critical Illness Score, VIF = variance inflation factor.

### 3.3. Logistic multifactorial analysis of the occurrence of nutritional substandard in critically ill patients

As a result of logistic regression analysis, mNU-TRIC score, catecholamines, feeding intolerance, and APACHE II score were found to be the risk factors for the occurrence of nutritional substandard in critically ill patients (OR = 3.690, 3.750, 5.194, and 1.249, 95% CI = 2.233–6.298, 1.526–9.215, 1.684–16.027, 1.146–1.360, *P* < .05), and GCS score was a protective factor for the occurrence of nutritional substandard in critically ill patients (OR = 0.760, 95% CI = 0.675–0.856, *P* < .05), as shown in Table 3.

**Table 3:** Logistic multivariate analysis of the occurrence of malnutrition in severe patients.

Influencing factor	*B* value	SE	Wald *X*²	*P*	OR value	The OR value was 95% CI
GCS score	−0.275	0.061	20.477	<.001	0.760	0.675–0.856
mNU-TRIC Score	1.306	0.256	25.962	<.001	3.690	2.233–6.298
Catecholamines	1.322	0.459	8.303	.004	3.750	1.526–9.215
Feeding intolerance	1.648	0.575	8.215	.004	5.194	1.684–16.027
APACHE II Score	0.222	0.044	25.896	<.001	1.249	1.146–1.360

APACHE II = Acute Physiology and Chronic Health Evaluation II, GCS = Glasgow Coma score, mNU-TRIC = modified Nutritional Risk in Critical Illness Score.

### 3.4. Column line diagram construction

**Figure 1.**
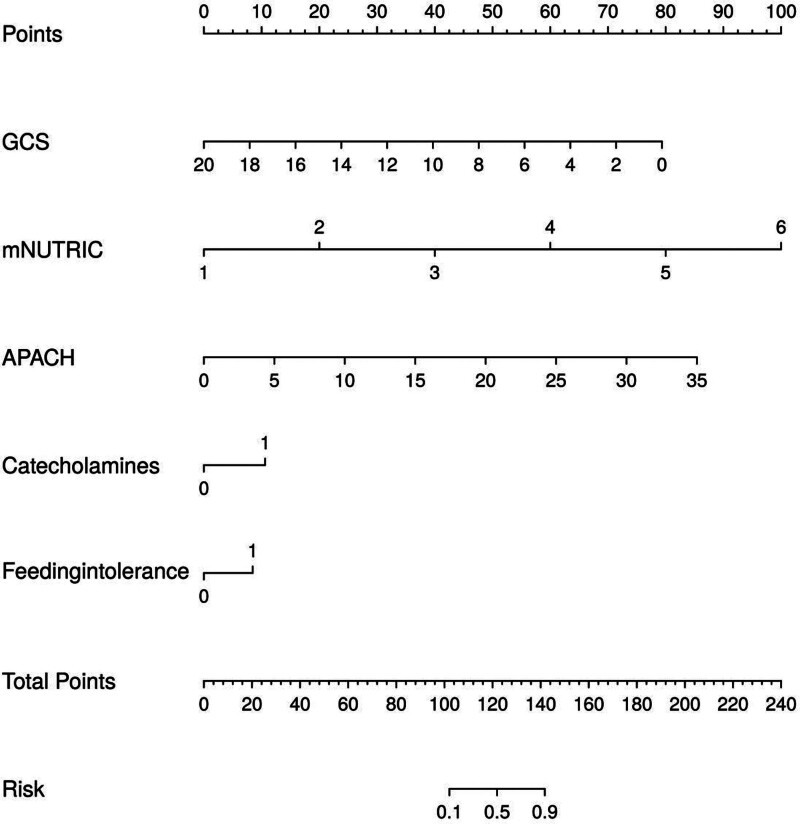
Column diagram construction.

According to the results of multifactor logistic regression analysis, the 5 independent influencing factors were used as predictors and the corresponding column line diagrams were drawn to visualize and individually assess the incidence of patients’ nutritional substandard, and the total sum of the prediction point scores corresponding to each variable corresponded to the bottom scale of the column line diagrams, as shown in Figure 1.

### 3.5. Internal validation curve plotting

**Figure 2.**
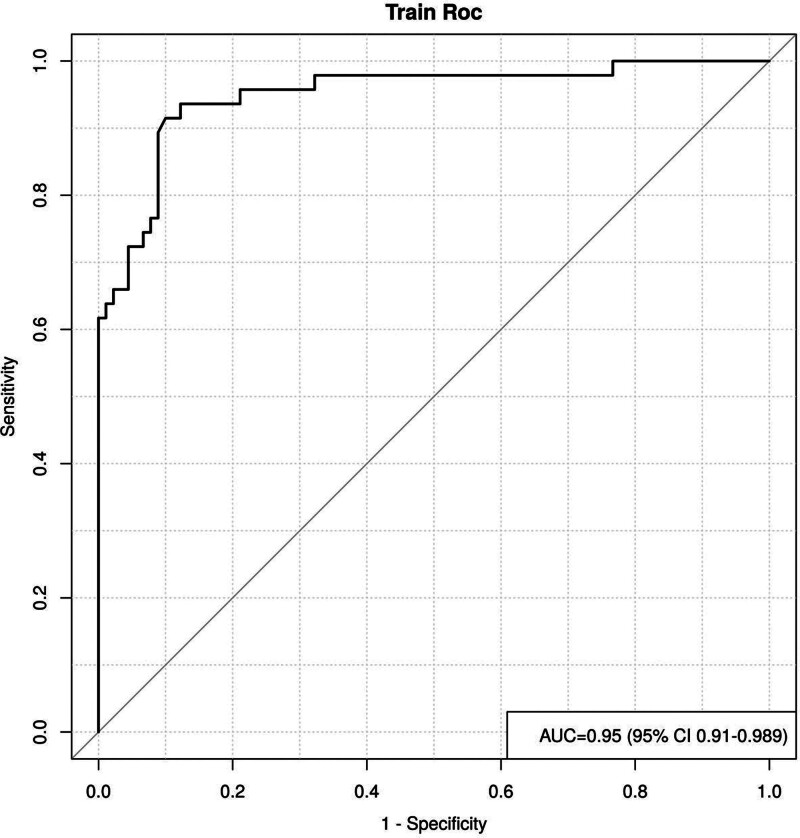
Internal verification curve drawing.

The ROC curve was plotted for internal validation, and the results showed an area under the curve value of 0.950 with a 95% CI of (0.910–0.989), indicating that the overall accuracy of the prediction model is high and there is a high degree of consistency with the actual risk, see Figure 2.

### 3.6. Comparison of nutritional status and enteral nutrition tolerance

The rate of enteral nutrition intolerance in patients after implementation (3.73%) was lower than that before implementation (11.68%), and the difference was statistically significant (*x*² = 6.155, *P* < .05), which indicates that the enteral nutrition care model based on evidence-based medicine can effectively reduce the intolerance of enteral nutrition in patients, see Table 4.

**Table 4:** Comparison of enteral nutrition tolerance before and after implementation.

Group	n	Intolerance	Tolerate
Before implementation	137	16 (11.68)	121 (88.32)
post-implementation	137	5 (3.73)	132 (96.27)
*x²*	–	6.155	
*P*	–	.013	

**Figure 3.**
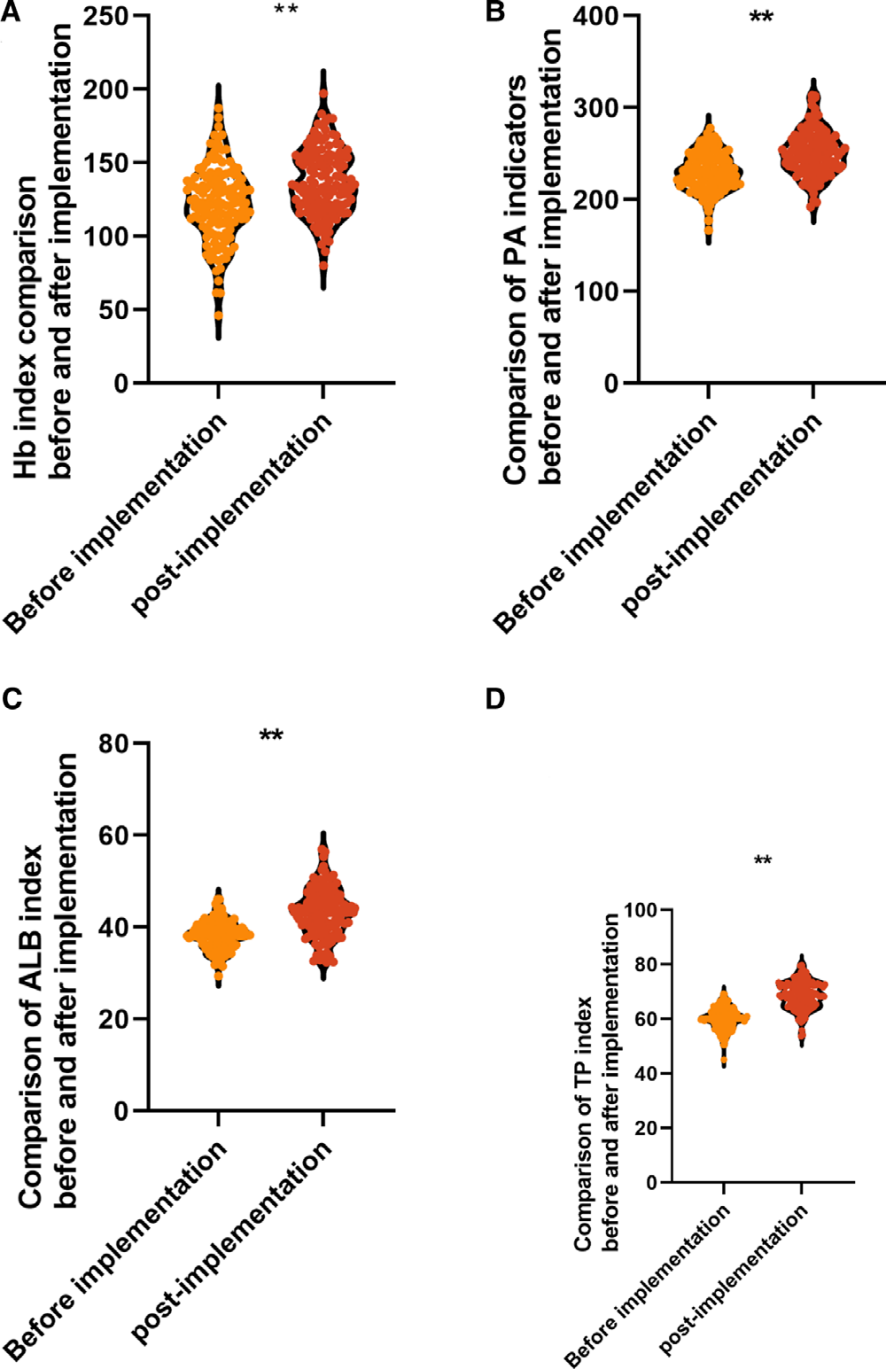
Comparison of nutritional indexes before and after implementation. Note: ALB is albumin, TP is total protein, PA is prealbumin, and Hb is hemoglobin; * is *P* < .05 and ** is *P* < .001.

ALB, TP, PA, and Hb are all important nutritional indicators, effectively reflecting the nutritional status of the organism, and after implementation, ALB (68.73 ± 5.02 vs 60.12 ± 4.05) g/L, TP (43.16 ± 5.13 vs 38.15 ± 3.07) g/L, PA (250.73 ± 24.01 vs 230.24 ± 20.01) mg/L, and Hb (137.15 ± 22.03vs.122.43 ± 24.98) g/L were higher than those before implementation, and the difference was statistically significant (*P* < .05), thus indicating that the enteral nutrition care model based on evidence-based medicine can effectively improve the nutritional status of patients, see Figure 3.

## 4. Discussion

Critically ill patients often appear a series of physiopathological and metabolic changes, coupled with a part of the patient cannot eat normally, energy intake is reduced, enteral nutrition support is particularly important in the treatment of critically ill patients, but the global ICU is still common enteral nutrition substandard problem, seriously affecting the prognosis of critically ill patients.^[19,20]^ Therefore, actively analyzing the relevant factors affecting the substandard enteral nutrition of critically ill patients and adopting scientific and effective measures are the key to improving the clinical prognosis of patients.

Catecholamines are neurotropic substances containing catechol and amine groups, which are bound through the enzymatic step of L-tyrosine at the location of sympathetic nerves, adrenal medulla, and chromaffin cells; feeding intolerance is a series of adverse reactions that occur when the digestive system of the patient who is retreated with enteral nutrition is unable to effectively digest, absorb, and tolerate the nutrients given.^[21,22]^ The data in this study showed that the rate of catecholamine use and feeding intolerance in the non-attainment group was higher than that in the attainment group, and all of these indicators were independent risk factors for nutritional non-attainment in critically ill patients (*P* < .05). The reason may be that critically ill patients usually have hemodynamic instability, the use of catecholamines can maintain hemodynamic stability, but may aggravate intestinal ischemia as well as reduce the intestinal nutrition tolerance, in line with the argument of Möller et al.^[23]^ And through the recommendation of the joint guidelines of American Society for Parenteral and Enteral Nutrition and society of critical care medicine, it was found that enteral nutrition is started in critically ill patients who are admitted to the ICU with successful fluid resuscitation or hemodynamically stabilized by the application of vasoactive medications for 24 to 48 hours, but it should be discontinued when they receive high doses of catecholamines.^[24]^ Relevant data indicated that, on the one hand, catecholamines can constrict blood vessels, leading to intestinal ischemia, which can damage the integrity and function of the intestinal mucosa, leading to intestinal obstruction or intestinal paralysis, reducing the speed of intestinal content transport, interfering with the release and action of intestinal endocrine hormones, and decreasing the absorption of nutrients; on the other hand, catecholamines may interact with other medications, increasing the risk of gastrointestinal complications, and affecting the On the other hand, catecholamines may interact with other drugs, increasing the risk of gastrointestinal complications, affecting nutrient absorption or metabolism, and thus increasing the risk of enteral nutritional substandard development. According to Regmee et al,^[25]^ feeding intolerance usually manifests itself in diarrhea, vomiting, abdominal distension, etc, resulting in a shortened retention time of nutrients in the intestine, and even if adequate enteral nutritional supplementation is carried out, the actual amount of absorption is insufficient, and the nutrient intake decreases, resulting in a higher risk of nutritional substandardization. Previous reports indicated that patients with feeding intolerance need to reduce the speed or concentration of enteral nutrition infusion in combination with the actual situation, or even suspend the infusion to alleviate the intolerance symptoms, which directly leads to a reduction in the nutritional intake of the patient, and fails to meet their high metabolic demand, resulting in nutritional substandard.^[26]^ Therefore, clinical caregivers should pay full attention to patients using catecholamines to maintain hemodynamics, be alert to the occurrence of feeding intolerance, and take timely measures to improve patients’ clinical outcomes.

The GCS score was published in 1974 by Graham Teasdale and Bryan J. Jennett, 2 professors of neurosurgery at the University of Glasgow, as an important indicator for medical staff to evaluate the severity of the patient’s condition, and also as a predictor of the patient’s functional recovery; the mNU-TRIC score is an important indicator for the assessment of the nutritional status of the critically ill patient; the APACHEⅡ score is widely used in ICUs. score is a widely used condition assessment system in ICUs.^[27,28]^ The data in this study showed that there was a significant difference between the GCS score, mNU-TRIC score, and APACHE II score of the 2 groups of patients in the non-attainment group (*P* < .05), and that all of the above indexes were independent influences on nutritional non-attainment in critically ill patients (*P* < .05). Shu et al^[29]^ stated that the higher the APACHE II score reflected the more severe condition, and each increase of 1 in the score increased the risk of non-attainment by 1 in the risk of non-attainment. Score increased the risk of nonattainment by 1.111-fold, and the risk of nonattainment increased accordingly in patients with comorbidities, suggesting that intestinal function and tolerance are affected by the overall condition. Groups of critically ill patients with high APACHE II scores are usually in a state of stress, hemodynamic instability, the body tissue to ensure the blood supply of the heart, liver, and other important organs and tissues, which will cause a decrease in intestinal blood flow, which in turn will damage the intestinal function, the patient in the receipt of enteral nutrition, gastrointestinal dysfunction, nutrient absorption is reduced, and increase the risk of nutritional non-attainment. And refer to Ma et al^[30]^ scholars found that some seriously ill patients due to the disease caused by coma or unconsciousness and other factors, the need for long-term restriction of bed rest, reduced activity, intestinal peristalsis slowed down, may increase abdominal distension, gastric retention, dyspepsia, and other symptoms occur, leading to enteral nutrition to slow down or even pause, the amount of feeding increased, and therefore disturbed, the risk of nutritional failure to meet the standard occurred to rise. mNU-TRIC a high score suggests that the organism is at high nutritional risk, and the severity of the disease is higher, and there may be organ failure, infections, etc, which affects intestinal function, leading to intestinal mucosal damage, intestinal dyskinesia, intestinal bacterial translocation, etc, which affects absorption of nutrients and leads to nutritional substandard, which is further corroborated by the results of the study conducted by Pannu et al.^[31]^ Column line graph is an intuitive and effective prediction tool, which plays an important role in medical research and clinical practice, in this paper, we can observe the risk of nutritional substandard occurrence in critically ill patients by using the column line graph, and the levels of its indicators change with the probability of the occurrence of the risk; and the ROC curve is a graphical tool to assess the performance of classification models, in this paper, we show the performance of each indicator in predicting the occurrence of nutritional substandard in critically ill patients by plotting ROC curves, and calculate AOC curves to show the performance of each indicator. In this paper, the performance of each index in predicting the occurrence of nutritional substandard in critically ill patients was demonstrated by plotting the ROC curve graph and calculating the area under the curve value to assess the accuracy of the prediction model. This suggests that enteral nutrition support in critically ill patients should be combined with the actual situation of the patient, and targeted measures should be taken to improve the body’s nutrition and improve the intolerance of the patient.

The results of this study show that all nutritional indicators of patients after the implementation of the evidence-based medicine enteral nutrition care model were higher than those before the implementation, and the rate of enteral nutrition intolerance was lower than that before the implementation (*P* < .05), which indicates that the evidence-based medicine enteral nutrition care model has a positive impact on the nutritional status of critically ill patients and enteral nutrition tolerance, which is in line with the argument of Mazzini et al.^[32]^ Evidence-based philosophy refers to the philosophy and methodology of integrating the best research evidence, clinical expertise, and patient values and preferences to make clinical decisions in healthcare, education, and social work, emphasizing a dynamic, critical decision-making process, while the core idea of evidence-based medicine is to integrate clinical evidence, personal experience, and the actual condition and wishes of the patient in healthcare decision-making.^[33]^ In this study, an evidence-based enteral nutrition care model was implemented for critically ill patients, through which the gastrointestinal function of patients was assessed, appropriate feeding methods and formulas were selected for early enteral nutrition to reduce complications; personalized nutritional program development was performed by combining patients’ nutritional status, disease status, and tolerance ability, etc, assessing the actual nutritional needs of the patients, and selecting enteral nutrition formulas for providing to the patients and making adjustments according to the patients’ Real-time situation to make adjustments to ensure that the patient’s nutrients are fully absorbed; and follow the principle of gradual feeding, to avoid the rapid increase in the volume and concentration of nutrient solution, and closely observe the patient’s tolerance situation, to ensure that the body has sufficient nutrition for consumption at the same time, to reduce the rate of feeding intolerance, to improve the nutritional status of the body, and to improve the prognosis. And refer to Lin et al^[34]^ who study can be seen to further support the results of this study. Evidence-based nursing measures emphasize the importance of nutritional preparation formula management, improve the tolerance of enteral nutrition support, reduce the number of interruptions of enteral feeding, and ensure the ability to supply nutrients.

## 5. Conclusion

In summary, there are more relevant factors affecting the occurrence of nutritional substandard in critically ill patients, including GCS score, mNU-TRIC score, catecholamines, feeding intolerance, and APACHE II score, and constructing a risk prediction model by using the above indexes can effectively screen high-risk groups, and the evidence-based medicine-based enteral nutrition care model has a positive effect on the nutritional status of critically ill patients and enteral nutrition tolerance, which is worth popularizing.

## Author contributions

**Conceptualization:** Ya Liu.

**Data curation:** XiaoXia Ye.

**Formal analysis:** Jie Wang, Qingqin Lu.

**Investigation:** Qingqin Lu.

**Methodology:** Jianhui Wang.

**Project administration:** XiaoXia Ye.

**Supervision:** Jie Wang.

**Visualization:** Jianhui Wang, Ya Liu.

**Writing – review & editing:** Jie Wang, Ya Liu.

**Writing – original draft:** Jianhui Wang, XiaoXia Ye, Qingqin Lu.

## Supplementary Material
